# A Tribute to Dr Gholam A Peyman

**Published:** 2011-01

**Authors:** Masoud Soheilian

**Affiliations:** Ophthalmic Research Center, Shahid Beheshti University of Medical Sciences, Tehran, Iran

Gholam A Peyman is an outstanding and brilliant mentor, educator, scientist, and surgeon.

**Figure:**
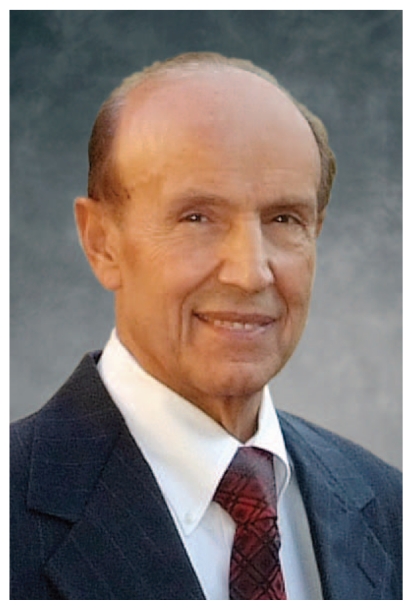


Dr Peyman was born in Shiraz, Iran and upon finishing high school, moved to Germany . He completed medical school, a residency in ophthalmology and a fellowship program in vitreoretinal diseases. After finishing his fellowship, Dr Peyman sought further training in the US. During Dr Peyman’s training as a fellow in vitreoretinal disease at the Jules Stein Eye Institute, affiliated to the University Of California School Of Medicine, Los Angeles (UCLA), he was offered a position as Assistant Professor. After a meeting with Dr Morton Goldberg he was recruited for a position at the University of Illinois, Chicago, where he was a faculty member until 1987. He then moved to New Orleans where he served as the Chief of Vitreoretinal Surgery at Louisiana State University Eye Center and then at Tulane University. Throughout his career in Chicago and New Orleans, his research was constantly decorated with spectacular accomplishments and he received numerous grants from the NIH, and various foundations and pharmaceutical companies. In 2001, after hurricane Katrina he relocated to Arizona where he joined the faculty of the University of Arizona, Tucson, as Joint Professor of Optical Science & Engineering. In 2009, he finally retired from Tulane Medical School, however he did not give up his academic activity and showed that retirement was not the end of his career, it was actually a new life. This time, he was assigned as Professor of Biomedical Sciences at University of Arizona, Phoenix.

Dr Peyman’s achievements are outstanding. He has authored 879 scientific papers, 10 books, and 4 book chapters during his accomplished career that spans through 40 years and still continues today. He is a member of the editorial board of 9 distinguished ophthalmology journals. He has received the most prestigious of awards and achieved the highest of accolades including the Lifetime Achievement Award of the American Academy of Ophthalmology and inclusion in the American Society of Cataract and Refractive Surgery (ASCRS) Hall of Fame.

Dr Peyman conducted pioneering studies in intraocular drug delivery, and refractive and vitreoretinal surgery. He established the techniques of eye-wall resection and endoresection for intraocular tumors, and was the first to perform a retinochoroidal biopsy and transplant retinal pigment epithelial cells for age-related macular degeneration. He is also a pioneer in photodynamic therapy.

Dr Peyman’s innovative investigations have resulted in 120 US patents, the most for any ophthalmologist. He described the first pressure-controlled valve (the Krupin valve) for glaucoma surgery, and with Dr Koziol, he developed the first telescopic IOL for patients with macular disease. He was also among the first to implant an artificial silicone retina in patients with retinitis pigmentosa.

Perhaps the most recognized work was a patent filed in 1985 for the use of excimer lasers for correction of refractive errors by stromal ablation under a corneal flap, the currently well-known procedure called LASIK.^1^

Throughout his career, Dr Peyman was a superb teacher of medical students, residents, and fellows. He was a doctor’s doctor. He was respected by his colleagues and treasured by his patients for his warm and caring manner.

Dr Peyman’s academic work, innovations, research, and the professional training of over 200 fellows from all over the world, have directly and indirectly saved the sight of millions of people.

The 20^th^ Iranian Congress of Ophthalmology, held in Tehran in November, 2010, had the honor to have Dr Peyman as a guest speaker in his homeland and to pay tribute to his pioneering work and accomplishments. In addition, Dr Peyman was awarded the first gold medal of the Iranian Society of Ophthalmology.

The Iranian Society of Vitreoretinal Surgeons, with endorsement of the Board of Trustees of the Iranian Society of Ophthalmology, has decided to honor the fruitful career and achievements of Dr Peyman by hereafter holding a lecture in his name annually. This year, we were fortunate to arrange the first “Peyman Lecture” presented by Dr Peyman himself, entitled “Combination Therapies in Ophthalmology: Implications for Intravitreal Delivery”, which has been featured in this issue of the Journal of Ophthalmic & Vision Research.^2^
